# Propagating Surface Plasmon Polaritons on Systems with Variable Periodicity and Variable Gap-Depth

**DOI:** 10.3390/ma13214753

**Published:** 2020-10-24

**Authors:** Silas O’Toole, Dominic Zerulla

**Affiliations:** School of Physics, University College Dublin, Dublin 4, Ireland; silas.o-toole@ucdconnect.ie

**Keywords:** SPP, Surface Plasmon Polariton, tuneable, active plasmonics, variable gap

## Abstract

Here we report on both simulations and experimental results on propagation and transmission of Surface Plasmon Polaritons (SPPs) through tunable gaps which were initially motivated by excitation of SPPs on a periodic arrangement of nanowires with mechanically tuneable periodicity. The general ability to vary the two-dimensional lattice constant results in an additional degree of freedom, permitting excitation of SPP’s for any combination of wavelength and angle of incidence within the tuning range of the system. Fabrication of the tunable system includes a transition from a continuously metal coated surface to small metal ribbons which can be separated from each other as a result of mechanical strain applied to the flexible PDMS substrate. This also results in the creation of tuneable gaps between the metal ribbons and variations in the thickness of the metal coatings. In order to explain the propagation of SPPs through such gaps we have employed Finite Difference Time Domain (FDTD) simulations of SPPs through model systems which contain gaps with varying depths and metal fillings.

## 1. Introduction

Planar waveguides and photonic crystal structures are being intensively investigated as primary solutions for integrated photonic devices. However, there may be an alternative approach to the manufacturing of highly integrated optical devices with structural elements that are smaller than the wavelength, which nevertheless enables strong guidance and manipulation of light—the use of metallic and metallodielectric nanostructures in conjunction with Surface Plasmon Polaritons (SPPs). This approach is now branded as “the next big step” in nanotechnology. Here, we present an adaptive material that consists of metallic ribbons on a tuneable polymer substrate.

SPP’s are mixed states of photons and electron density waves that propagate along the surface of a conductor. The interaction of light with matter in nano- and mesostructured metallic interfaces has led to a new branch of photonics called plasmonics [1]. Today, SPP’s play an important role in the fundamental understanding of quantum behavior at nano- and meso-scales [2,3,4,5,6], as well as in the development of novel spectroscopic techniques, such as Surface Plasmon Resonance (SPR) [7,8] and ultra surface sensitive methods, from Surface Enhanced Raman Scattering (SERS) on nanostructured surfaces [9,10] to subwavelength optics [11].

The excitation of SPP’s cannot be achieved by direct irradiation of photons on a smooth metal surface, but it requires special optical configurations, including those that were initially proposed by Raether and Kretschmann [12], and Otto [13]. A common property shared by these setups involves photons progressing from an effectively optically denser medium before impinging on the metal in an attenuated total reflection (ATR) setup. However, SPP’s can be excited on periodically modulated metal surfaces as a result of an exchange of the k vector between the surface and the impinging photons, given by the reciprocal lattice constant 2π/d of the periodic structure [12,14,15,16,17,18].

We introduce a plasmonic system with tuneable periodicity in order to motivate the following investigations [19,20,21]. The ability to choose properties of the incident SPP is enabled by this development and provides one of the pivotal building blocks in creating active plasmonic devices. The additional degree of freedom in exciting SPP’s, granted by such a tuneable system, has been already investigated in an article in 2007 [20]. Instead, we are focusing on the propagation properties of SPPs through such tuneable systems, i.e., the transmission of SPPs through variable gaps, instead. To facilitate a clear understanding of all phenomena involved, we are introducing simplified model systems, which consist of a tuneable gap that is additionally tuneable in its depth. To analyze the SPP’s behaviour, we employ Finite Difference Time Domain simulations (MEEP) to deeply investigate the propagation of SPP’s through these gaps on both interfaces air/metal and dielectric/metal. Building on the publication [20], we are focussing on the gapped nature of the tuneable system with fully featured three-dimensional MEEP derived FDTD simulations to thoroughly investigate the plasmonic behaviour employing a realistic non-plane wave light source and high spatial resolution mesh grid.

Propagation of SPP’s through three-dimensional more complex, but non tuneable structures (including three-fold symmetric structures) have also been investigated [22,23,24,25].

## 2. Results

**Figure 1:**
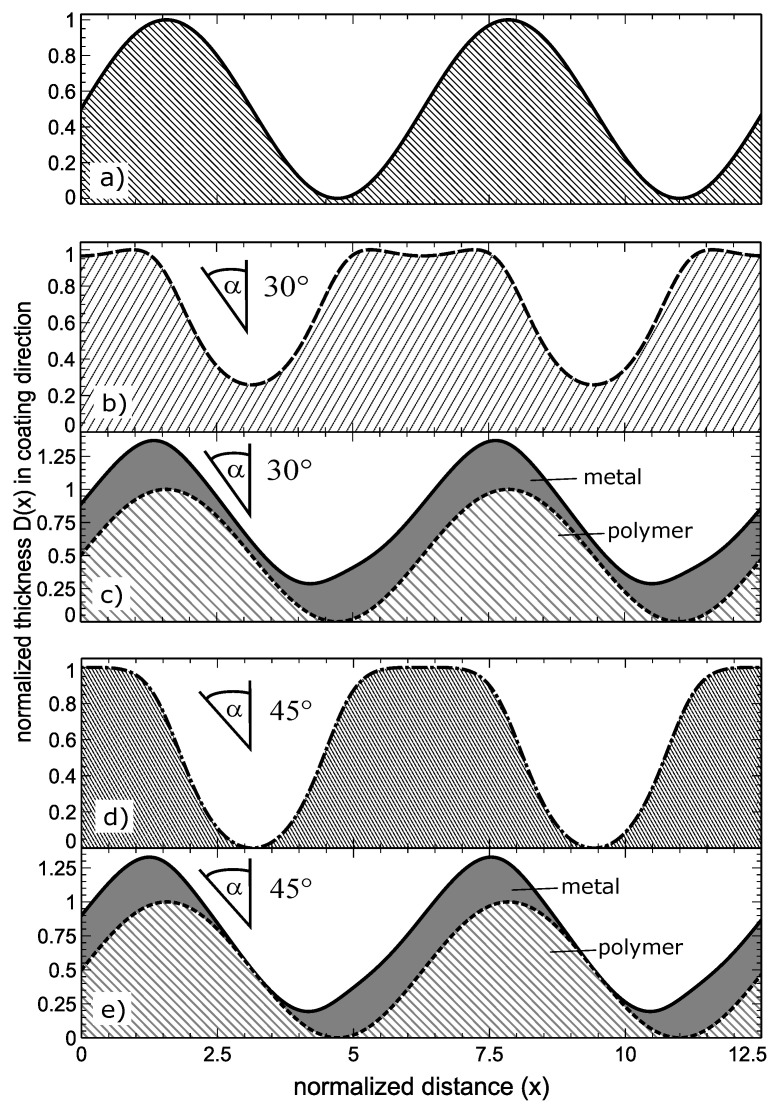
Visualisation of the thickness of a metal coating when evaporating metal on a (**a**) sinusoidal PMDS profile at 30 degrees; (**b**) coating thickness and (**c**) coating plus sinusoidal profile and 45 degrees; (**d**) coating thickness and (**e**) coating plus sinusoidal profile.

The manufacturing of the desired tuneable structures is a multistep procedure described in great detail in the supplement of F. Katzenberg’s article [19]. In brief, polydimethyl siloxane (PDMS) is cast to an approximately 1 mm thick rubber film. A uni-axial strain is then applied to the sample while an argon plasma truncates and crosslinks the upper surface of the film. This leads to relaxation processes in this thin surface region due to the fragmentation of macromolecular segments and renewed crosslinking. After the strain is removed, the lower non-relaxed part of the sample shrinks and causes compressive stress to the upper relaxed part. This produces sinusoidal ripples at the surface in order to compensate for the consequent length differences between the two layers. The sinusoidal profile is subsequently silver coated at an oblique angle of typically 30${}^{\circ }$–45${}^{\circ }$ in the plane perpendicular to the grooves, which results in a thickness modulated silver film at the surface, as visualized in Figure 1.

**Figure 2:**
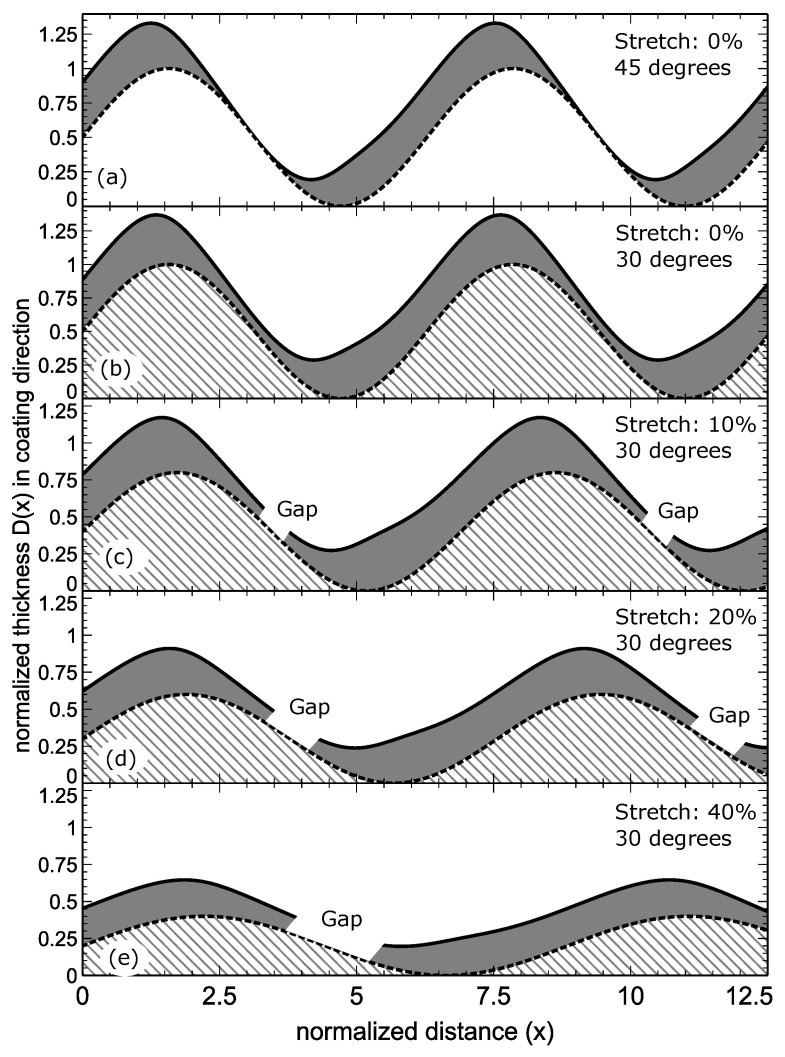
Visualisation of the thickness of a metal coating when evaporating metal on an sinusoidal PMDS profile at (**a**) 45 degrees and (**b**–**e**) 30 degrees. Additional we demonstrate the effect of the strain which from (**c**–**e**) produces gaps at the weakest part of the metal film and additionally is causing a flattening of the sine profile and consequently of the metal ribbons in contact with the PDMS substrate. Most importantly, the system has created a metallic system with reversibly tuneable gaps.

Re-application of strain leads to fractures along the thin metal regions parallel to the grooves. Such a process is illustrated in four steps in Figure 2. The reason for the formation of gaps subsequent to strain beyond a threshold is because of the strongly varying thickness of the metal coating. In the presented example the metal was coated at 45 degrees against the surface normal in the plane perpendicular to the sinusoidal grooves of the substrate. For geometric reasons, this leads to a modulation of the thickness of the metal film D(x), depending on the slope of the sinusoidal surface corrugation in comparison to the incoming angle, an incidence parallel to the surface corrugation’s normal results in maximum coating thickness, striving incidence in minimum thickness, as shown in Equations (1) and (2):

(1) $$\begin{array}{c} D\left(x\right)=D_{0}\cos\left(\alpha -\beta \left(x\right)\right) \end{array}$$

(2) $$\begin{array}{c} \beta (x)=\arctan(\cos(x)) \end{array}$$

with $D_{0}$ = equivalent coating thickness at normal incidence, α = coating angle against normal, β(x) = angle against surface normal in point (x). To illustrate Equation (1)/(2), the resulting effective thicknesses are plotted in Figure 1b,d.

Evaporation of a metal onto a sinusoidal profile in a plane that is perpendicular to the grooves at an oblique angle of 30 degrees is already causing severe alterations of the local thickness from 100% to below 30% see Figure 1c and Figure 2b. Please note in this regard that the real slope of a sinusoidal surface depends on its aspect ratio. For the simulations, we have used normalised sine functions (unit circle) that reach a maximum slope of 1.

**Figure 3:**
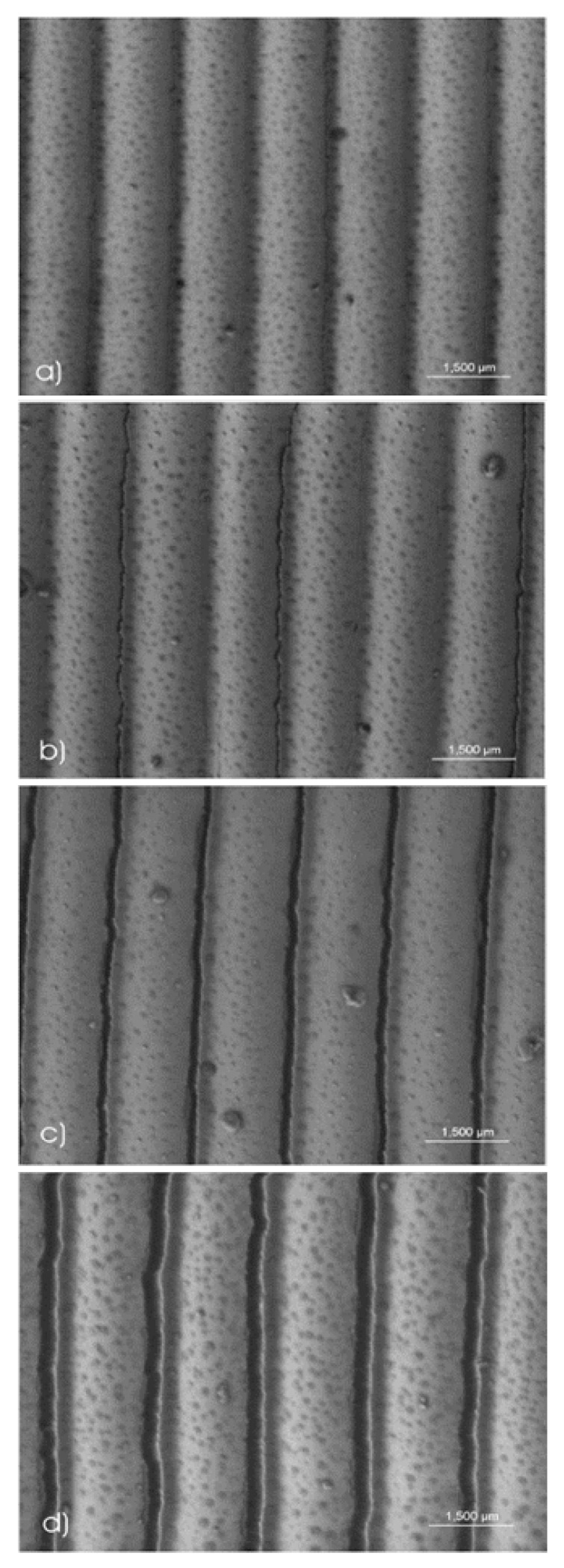
Scanning electron micrograph of a silver coated periodic polymer structure with average silver thickness of 50 nm: (**a**) unstretched, periodicity 750 nm; (**b**) 5% stretched (first gaps appear); (**c**) 10% stretched (all gaps appear); (**d**) 20% stretched, periodicity 900 nm.

The system that we experimentally realized, see Figure 3 below, has a smaller maximum slope hence it is showing less severe shadowing effects. In fact, using a norm sine function would cause thicknesses down to 0 nm if evaporated with an angle at 45 degrees, as demonstrated in Figure 2a and would create broader non coated regions at even more glancing angles. Note that the regions of lowest thickness are quite localized, creating an effective hyphenation when applying strain beyond a threshold that is capable of breaking the metal film at its thinnest points (Figure 2c–e and Figure 3b–d). The widths of the metal ribbons remain almost unchanged during the strain process. Stretching the substrate results in a widening of the sharply defined gaps.

Subjecting the metal coated sinussoidal surface to strain beyond a certain threshold results in all grooves becoming broken. The resulting metallic ribbon structures are not perfect, but, when averaged over larger areas, are sufficiently parallel to each other. Spacing, and hence fundamental periodicity of the grating, depend linearly on the applied strain. The separation of the metallic wires can be reversibly adjusted thus allowing for dynamical changes in the grating constant. The initial value of the grating depends on the chosen PDMS parameters, the stretching prior to the argon plasma treatment and the plasma parameters. This procedure so far allows periodicities between 350 nm and 2 μm, while the separation can be fine-tuned from 0 to above 50 percent of the initial periodicity, resulting in gaps from few nanometers to a micron. Figure 3 shows a step by step series of scanning electron micrographs of the crucial steps in the formation and tuning of gaps.

**Figure 4:**
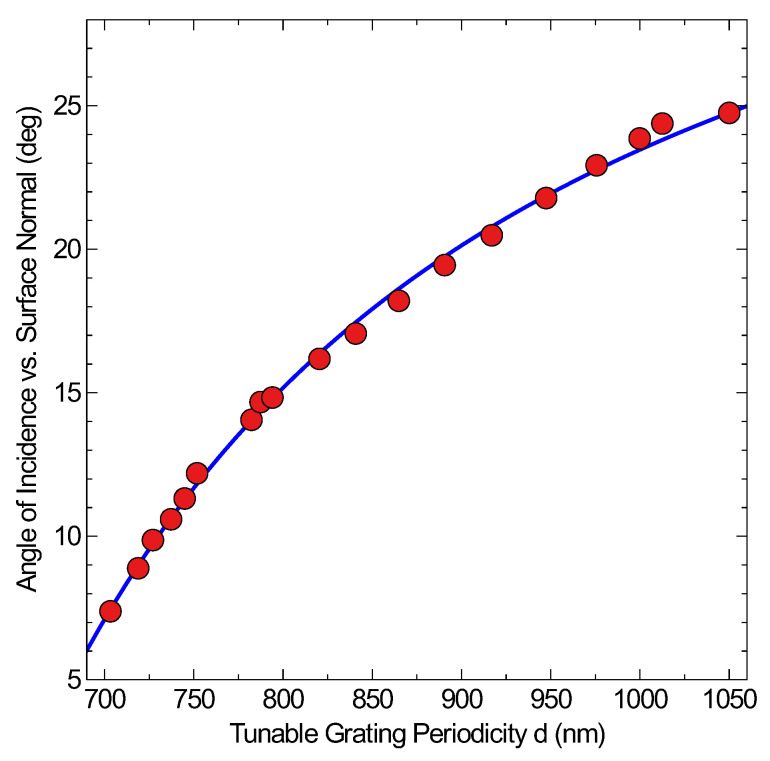
Experimental results of the tuneable gap system, demonstrating the capability to adapt while maintaining Surface Plasmon Polaritons (SPP) conditions. The fitting curve is based on the momentum matching conditions of the SPP and the grating equation [20] following an arcsin behaviour. Further details are addressed in the experimental section.

The full tuneability of the system with respect to the angle of excitation is demonstrated in Figure 4, by changing the gap width over 300 nm resulting in an expanded tuneability of 20 degrees. Alternatively, the tunable systems provides the ability to excite SPPs at a wider range of wavelengths and thus this system becomes truly versatile showing the importance of tunable periodicity or modifiable gap widths which deserve a detailed investigation. To do this in a rigorous investigative manner, we set up simulations to probe the effect of varying both gap width and depth on plasmonic propagation through gaps in the silver.

**Figure 5:**
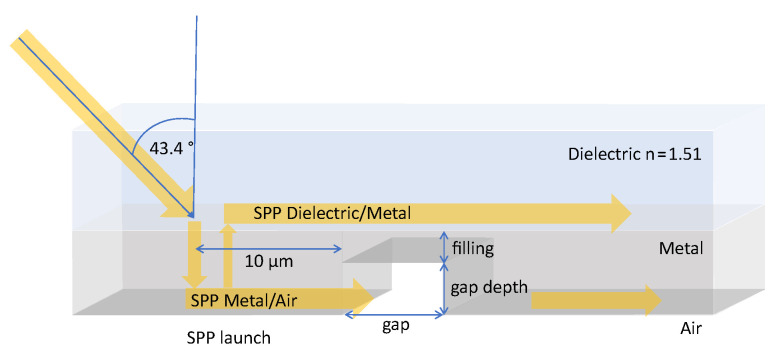
Setup of the simulation space. SPP Excitation area to the left.

In the following, we are introducing simplified model systems which permit us to analyze the plasmon propagation properties of such systems (see setup of the simulation space in Figure 5).

In order to separate the excitation effects from the propagation of the SPP, the system is using a dielectric coupler for excitation that is sufficiently spatially distanced (9.83μm) from the gap (left side in Figure 5) minimizing cross talk. The gap itself is an airgap which is tunable in its width but also additionally tunable in its depth. In order to allow for unhindered propagation of the SPP to the gap, we kept the metal thickness at its optimal value range of 45–50 nm but provided the gap with varying degrees of metal fillings. This enabled the analysis of the effect of a constriction in the metal film thickness in addition to its widths. The full simulation setup is sketched in Figure 5:

In our initial publication [20], we had demonstrated that such a tunable gap system can adapt to widely varying excitation wavelengths. Additionally, we could demonstrate that the transition from the initial sinusoidal surface without gaps (i.e., an uninterrupted metal surface) to a periodically interrupted surface (i.e., with gaps) does not change the reflectivity of such a system in an appreciable manner.

This is a bit surprising for two reasons:

- Firstly, SPPs, while highly damped and hence only equipped with a relatively short propagation length, should still be impeded by the discontinued metal surface in their propagation [5,6].
- Secondly, the sharp cracks in the surface, which are the gap boundaries, are excellent points of re-radiation, as their Fourier transform, i.e., spatial frequency response, is very broad.

However, as the simulations below will show, the electromagnetic fields will predominately provide coupling across the gap, after which the SPP can continue its propagation parallel to the interfaces.

In order to avoid misunderstanding, this article will focus on the transmission of SPPs through the gap and not on the gap modes themselves and their field strength, as many successful publications have already done [26].

**Figure 6:**
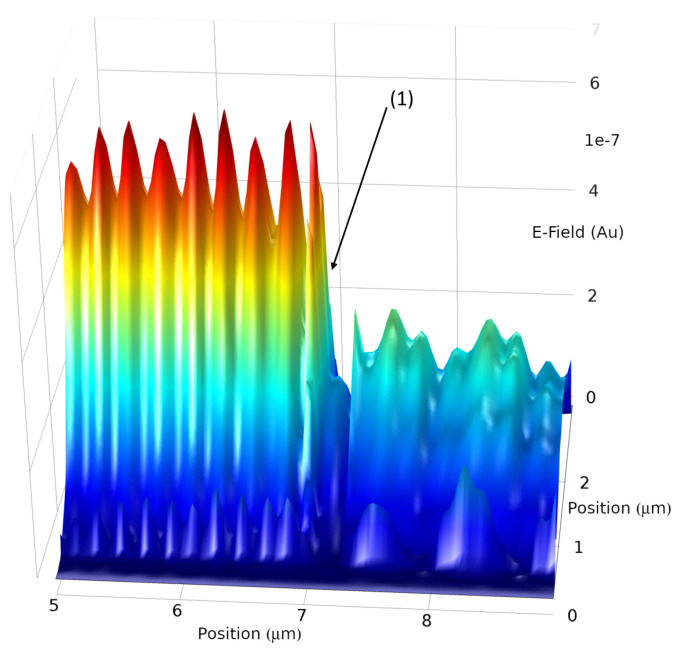
Three-dimensional (3D) Simulation, image is a representation in 3D of a two-dimensional (2D) plane in the near-field of the Metal Air interface showing the previously discussed data. X and *z*-axis are in microns, *y*-axis displays the E-Field strength.

**Figure 7:**
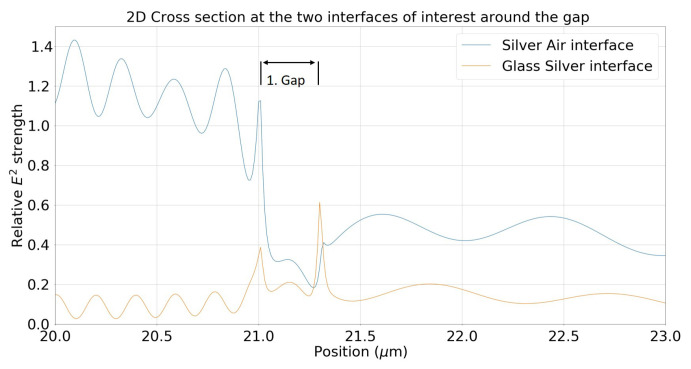
2D cross section for both interfaces for a gap width of 300 nm with no filling in the gap *x*-axis is position in the simulation in microns and the *y*-axis is E-Field strength relative to a comparable non-gap system.

**Figure 8:**
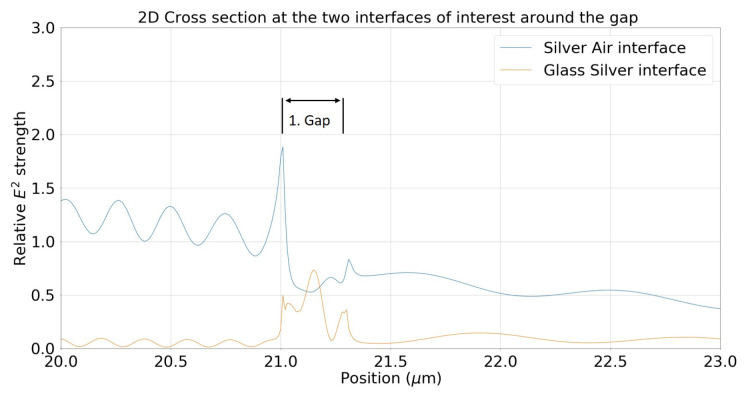
2D cross section for two interfaces at a gap width of 300 nm with a 30 nm filling in the gap. *x*-axis is position in the simulation in microns and the *y*-axis is E-Field strength relative to a comparable non-gap system.

Transmission and gap propagation was first studied by Maradudin et al. as early as 1983, which gave the original theoretical treatment of SPP propagation through gaps filled with various materials [27]. More recent experimental treatments of gap modes can be found by Seidel et al. [28] and cross coupling to separate modes were investigated by Sidorenko and Martin (glass/metal and metal/air) [29]. From the results of the gap sizes, certain features of SPPs are tunable, such as the energy at the start of the gap, the electric field of the plasmon after the gap, and the cross coupled wave mode. In the following, the metal used in the simulations is silver with constants from Johnson and Christy [30]. Without limiting the generality, we have chosen silver as a very strong plasmonic material for the visible spectrum. However, other commonly used plasmonic materials, such as gold, will not differ strongly regarding propagation of SPPs through gaps and are expected to follow the same trends. This article starts in visualizing the electric field distribution for a typical gap system with no filling in three dimensions. The metal air interface is directly excited and a plasmon launched parallel to the interface, this SPP is capable of exciting a second plasmon at the dielectric/metal interface that also propagates in the same direction. Both SPPs interfere strongly with each other, as demonstrated in Figure 6, Figure 7 and Figure 8.

**Figure 9:**
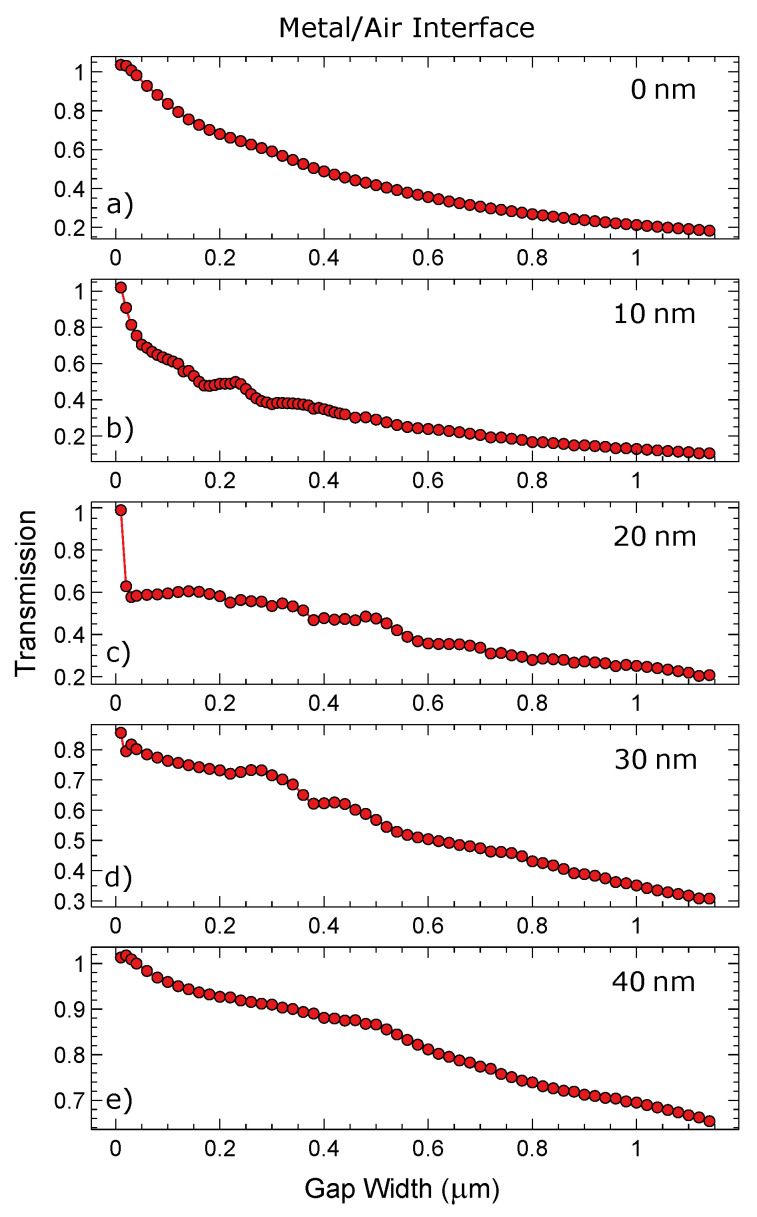
Transmission percentage with respect to gap width compared at the silver/air interface with a 10 nm resolution. X-axis is the gap width in nanometres and the *y*-axis is the transmission coefficient of the gap mode examined: (**a**) No Gap filling (**b**) 10 nm Gap filling (**c**) 20 nm Gap filling (**d**) 30 nm Gap filling (**e**) 40 nm Gap filling.

**Figure 10:**
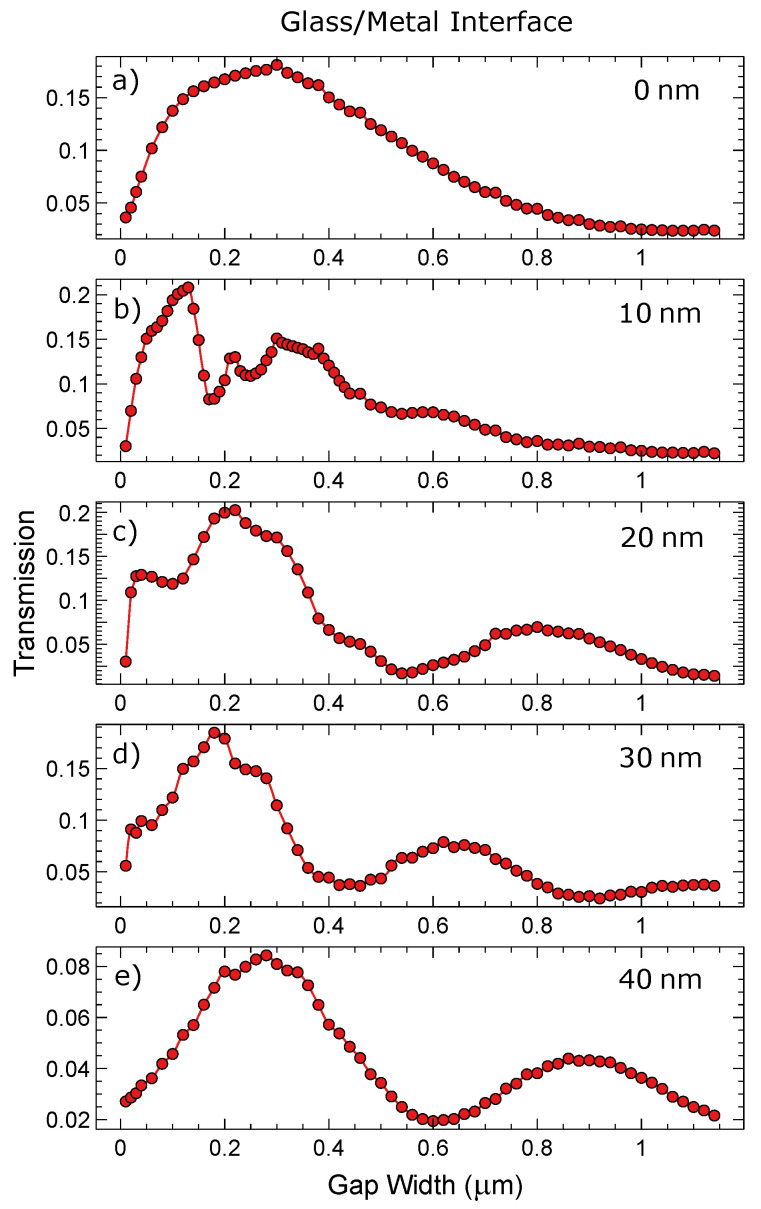
Transmission percentage with respect to gap width for various gaps compared at the glass/silver interface with a 10 nm resolution. X-axis is the gap width in nanometres and the *y*-axis is the transmission coefficient of the gap mode examined: (**a**) No Gap filling (**b**) 10 nm Gap filling (**c**) 20 nm Gap filling (**d**) 30 nm Gap filling (**e**) 40 nm Gap filling.

Figure 6 is the representation of a 2D cross-section of the ZX plane that originates from a full 3D simulation. The E${}^{2}$ intensity is represented as height and colour with arbitrary units and enabling comparison of the relative strengths. The above section runs from five to nine microns in the 3D simulation space on the *x*-axis with a size of 12×3μm in the full simulational space, this differs from the 2D simulation due to computation constraints. Care has been taken to avoid edge effect due to the space of such simulations. In Figure 6 the strong E-Field on the left is the plasmon, this interacts with the gap beginning at (1), which is 300 nm in width. The plasmon wave is partially reflected and causes interference with the incoming plasmon. Additionally there is a beating pattern visible on the left, which is due to a reflected mode on the glass/silver interface that interferes with the mode on the silver/air interface. Similar effects occur on the right side of the gap but are damped due to the SPP crossing the gap and a partial coupling to free radiation. The percentages of Transmission and coupling to the glass air interface can be seen in Figure 9 and Figure 10.

Figure 7 is also a 2D cross section of a single simulation for a fixed gap size of 300 nm. Displaying the data as a single line graph in contrast to the 3D image allows easy access for information extraction and direct comparisons of both modes. The figure displays both cross section of the silver/air interface (blue) and glass/silver interface (orange). The figure is a subset of a larger simulation focussing on the region of interest around the gap. The left side shows an incoming plasmon represented by the strong E-Field energy distribution (blue). The strong E-Field is visible until it reaches the gap at the relative position of 21 μm as depicted. The relative E-Field strength, as displayed on the *y*-axis, is a measure of plasmon energy relative to a point just before the same gap position but in a gapless setup. It is interesting to note that the energy can be higher than 1 due to the structure. There are two interference patterns, one from the reflected wave and another is a beating pattern from a mode that is present on the glass silver interface on the left side of the gap, which can be seen in the glass/silver interface (orange). There are also strong electric fields at the beginning and end of the gap owing to the sharp edge as to be expected. The standing waves on the other side of the gap are due to interference between the two modes, creating the pattern seen here. Care has been taken to make sure the simulation space is sufficiently large and with appropriate boundary conditions, so that back reflections at the end of the simulation space are not present. Similar interference patterns, as shown here, have also been observed experimentally in Seidel et al. [28].

In the following, we introduce gaps that are partially filled with the surrounding metal. To properly demonstrate some of the differences a metal filling makes to the E-Field around the gap there is an added cross section Figure 8 of the same sized gap but with a 30 nm filling inside the gap. The field distribution at the silver/air interface is relatively similar outside of a large increase in the electric field strength at the beginning of the gap. The beating pattern is also reduced quite visibly due to the lower amount of reflection, coupling weakly to the glass/silver interface as demonstrated. In Figure 8 on the glass/silver interface, the maximum of the electric field is near the centre of the gap with an energy distribution that indicates a low order gap mode forming, whereas, in Figure 7 (with no filling), the energy is mostly concentrated at the edges.

Figure 9 is investigating the relationship between gap width (*x*-axis) and the transmission of plasmonic energy across the gap. The data that are extracted from Figure 7 and Figure 8 are represented as a single point in Figure 9 and Figure 10. Calculations for the transmission percentages are described in the experimental section. All of the above calculations are taken at the silver/air interface and comprise the main plasmonic mode. As would be expected, there is a monotonic decrease in transmission percentage as the gap gets wider (with a max width at 1.14 μm). Each graph represents a gap with a different thickness of partial filling of silver. Increasing the filling improves the transmission allowing for fine tuning of the transmittance, even with large gap sizes. There are some minor deviations in the transmission % occurring in the partially filled gaps. These are due to increased or decreased cross coupling to the glass/silver interface from different gap sizes and resonance effects.

Figure 10 is similar to Figure 9, except that it is the glass/silver interface that is being investigated. The reference for the transmission % is the same as above. At this interface there is an additional loss pathway which dissipates light into the far field. Figure 9 and Figure 10 demonstrate that drastic change in transmission occurring at one interface do not strongly impact on the transmission on the other interface. Counter-intuitively the relative changes in transmission are more strongly seen on the glass/silver interface than on the silver/air interfere, as demonstrated in Figure 9 and Figure 10. As the filling increases there is a reduction in coupling to the glass metal interface and similarly with larger gaps leading to less coupling unless some resonance effects occur, as shown in the figure. At the metal air interface they are relatively smooth with only a few ranges of gap width indicating weaker resonance conditions. In contrast the glass/metal interface exhibits stronger gap width related modulations in the downward trend. The simulations were performed at different spatial discretization in order to ensure numerical stability of the presented results. For small gap sizes there could be additional pathways for coupling or transmission, such as tunnelling with more information on this topic found here [31,32].

## 3. Discussion

The simulations presented here confirm that the introduction of small gaps into an originally uninterrupted metal surfaces do not lead to significant alterations of the SPP propagation behaviour especially at the metal/air interface apart from losses that scale smoothly with the gap width. Instead of radiating widely into the far-field and, thus, changing the reflectivity of such systems, the energy is transmitted mainly in the original propagation direction. This is ideal for the here presented dynamically tunable systems that can respond to changes of the excitation wavelengths by changing their periodicity and hence their gap size without loosing their plasmonic properties. Furthermore, we have demonstrated how partial filling of the gaps with the surrounding metal (here silver) is causing changes to the transmission of SPPs. The gap depth is another parameter of particular importance, as it can be used to additionally fine tune the plasmonic behaviour in such systems, providing the designer with additional control of SPPs and towards active control.

The results presented here permit an outlook into the future, if we change our viewpoint—instead of focusing onto the gaps, we can investigate the metal fillings instead. These fillings can be regarded as constrictions of the cross section of the metallic structures which still permit SPPs to travel through as demonstrated above. Changing the filling results in changing of the cross section of the conductive metal. Applying a small voltage to the metal ribbons (e.g., on both ends of our simulation space) is causing a current to run through these constrictions. As the constrictions can be as low as 10 nm, as demonstrated here, the current density is strongly enhanced in these sections, leading to alterations of the optical constants of the materials. The rapid modulation of the current will lead to rapid modulation of the plasmonic behaviour—a strong platform for achieving active control. We have performed experimental and simulation based studies of this kind of active control, but, because of their volume and complexity, these investigations deserve their own publication. In summary, we have motivated the simulation studies with an experimentally realised tunable system that produces mechanically tunable gaps. Their behaviour was successfully analyzed by finite difference time domain simulations—leading to tunable systems with gaps and constrictions which open up a platform for new concepts of plasmonic active control.

## 4. Materials and Methods

MEEP or MIT Electromagnetic Equation Propagation is a free open source software package for the solving of electromagnetic simulations while using the FDTD methods. The FDTD method was chosen due to its versatility in application and ease of use. Finite difference methods have an advantage over other numerical methods in terms of their ability to solve time-evolving fields and for multiple frequencies in one simulation which is useful for realism of light sources used. The ability to easily manipulate the base code of this package to alter how some of the materials are modelled to suit newly available data was quite invaluable. The source used in these simulations is an oscillating charge that emits spherically and it is then attenuated using a Gaussian function. More information can be found on this in Oskooi et al. [33].

Simulations with larger memory requirements were completed at the Irish Centre for High-End Computing (ICHEC). For the high resolution or large simulation space, access to the supercomputing cluster was absolutely necessary. The high memory node in ICHEC was the main one used for these simulations. The High Memory nodes in ICHEC are a set of six nodes each containing 1.5 TiB of RAM, 2×20 core 2.4 GHz Intel Xeon Gold 6148 processors with 1 TiB of dedicated local SSD storage.

The overall experimental layout follows the below table with the following specific geometries. The top part of the simulation is covered in a glass block with input dimensions into MEEP of x = *∞* and y = 7.5 μm with centre at x = 0 and y = 1.25 μm. This is then coated with silver of thickness 50 nm and length of the simulation space (taking care to not have the silver overlap with the PML) including air gaps embedded into this layer. Below this is air of refractive index n = 1. The source is simply defined as the standard MEEP source with the position of the following

(3) $$\begin{array}{c} x=-4cos(\alpha )-6 \end{array}$$

(4) $$\begin{array}{c} y=4sin(\alpha )-2.5 \end{array}$$

with a similar directional “powerAmp” function so that the centre of the beam will be incident on the glass/silver interface at a position of x = −6 μm at the specified angle. A full list of important simulation parameters can be found in Table 1.

All of the transmission energies are in reference to a no gap system. All numbers are then presented as a percentage of a gapless system and not as a percentage of the whole plasmonic energy. Additionally for the gap simulations, each one is first time averaged over several periods of the incoming light, then the E${}^{2}$ energy is sampled at a point just after the gap (10 voxels, typically 100 nm depending on the simulation spatial resolution). Additionally, in order to avoid local beating patterns both interfaces have been integrating over a sufficiently large x size (1 μm). The reference points are at a dynamic distance as it changes with the gap size; however, the reference is also dynamic and changes in the same way thus leading to a fair comparison. The same method was applied for the glass/metal interface but using the same reference in a gapless system, so it is still a fraction of the same amount of electric field.

Subpixel averaging is a method applied in MEEP to smooth out interfaces so for several pixels at an interface the permittivity is a mixed value between the two materials rather than a sharp cutoff between the two, this may lead to issues in the lower gap sizes, where the minimum gap size (10 nm) is not a true gap due to this smoothing effect and, thus, the outlying data points.

The function used for fitting in Figure 4 is derived from the following simplified condition for a metal/air interface, as seen below:

(5) $$\frac{\omega }{c}\sin\left(\alpha_{inc}\right)+l\frac{2\pi }{d}\overset{!}{=}\frac{\omega }{c}\sqrt{\frac{\epsilon_{m}}{\epsilon_{m}+1}}$$

where $\epsilon_{m}$ is the real part of the metallic dielectric constant, c is the velocity of light in a vacuum, $\alpha_{inc}$ is the polar angle of incidence, ω is the angular frequency, l is an integer characterising the diffraction order of the light in the periodic structure, and d is the periodicity. This function can be rearranged with respect to the angle and thus lead to the following fitting function that was used.

(6) $$\alpha =c\arcsin(\frac{b}{x}+a)+d$$

where the exact constants are, a = 0.56132546285462, b = 277.55194089574, c = −58.023576269051, d = 81.252008567258.

The manufacturing of the desired tuneable structures is a multistep procedure described in great detail in the supplement of F. Katzenberg’s article [19]. In brief, polydimethyl siloxane (PDMS) is cast to an approximately 1 mm thick rubber film. A uni-axial strain is then applied to the sample, while an argon plasma truncates and crosslinks the upper surface of the film. For the SEM images of the prepared samples, a low-kV Hitachi S-4500 FEG scanning electron microscope (SEM) (operated at 1 kV to avoid electrical charging) was used.

## Figures and Tables

**Table 1:** Details regarding the simulation parameters used in MEEP.

**Resolution**	100 pixels per μm
**Size**	30 μm in X and 10 μm in Y
**PML Layer**	0.5 μm
**Wavelength**	532 ± 0.01 nm
**Material Glass**	Refractive index n = 1.51
**Material Silver**	MEEP inbuilt Johnson and Christy data
**Angle from normal**	43.4 degrees
**Time scale**	Length of 26 MEEP time units [33]
**Time averaging**	The averaging was done over 1 time unit split into 21 discrete steps
**Sub-pixel averaging**	On

## Abbreviations

SPP	Surface Plasmon Polariton
PDMS	PolyDiMethyl Siloxane
FDTD	Finite Difference Time Domain
